# Social inequalities in early exit from employment in Germany: a causal mediation analysis on the role of work, health, and work ability

**DOI:** 10.5271/sjweh.4043

**Published:** 2022-10-01

**Authors:** Max Rohrbacher, Hans Martin Hasselhorn

**Affiliations:** 1 Department of Occupational Health Science, School of Mechanical Engineering and Safety Engineering, University of Wuppertal, Wuppertal, Germany

**Keywords:** inverse odds weighting, longitudinal cohort, older worker, social inequality

## Abstract

**Objective:**

The aim of this study was to investigate the contribution of work factors, health, and work ability to social inequalities in early exit from employment among older employees in Germany.

**Methods:**

Longitudinal data from the representative German lidA Cohort study was linked with employment register data to obtain maximum information on exit routes out of paid employment. Information of N=2438 respondents, aged 46 and 52 at baseline, were obtained for a follow-up of six years (2011–2017). Causal mediation analysis with inverse odds weighting was conducted using discrete-time survival outcomes and baseline measurements of the socioeconomic status (SES: education), work factors, health, and work ability.

**Results:**

Older employees with low SES were at an increased risk of exiting employment early by receiving disability pension and through long-term unemployment but not through an unspecified labor market exit when compared to those with high and moderate SES. Low work ability accounted for up to 38% of the social inequalities in work exits into disability pension. Less-than-good physical health accounted for up to 59% of inequalities in work exits into long-term unemployment. Work factors contributed considerably to inequalities in exits through unemployment but not disability pension.

**Conclusions:**

This study finds social inequalities in early exits through disability pension and long-term unemployment among older employees in Germany, predominantly attributable to differences in work ability (disability pension) and physical health (unemployment). Investments in work ability and promotion of physical health may constitute promising approaches to counteract an increase of these inequalities.

In Germany, as in many other European countries, low-skilled workers drop out of the labor market earlier than highly-skilled workers (1). Recent pension reforms that aim to increase the statutory retirement age and sanction early exit routes out of paid employment will therefore affect this group more heavily, both in the short-term (eg, through loss of income) and the long-term (eg, through reduction in pension claims), when compared to those with higher socioeconomic status (SES) (2), thereby aggravating social inequalities in the work-retirement transition. For older employees with a lower SES, early exits from paid employment are rarely voluntary (1, 3) but may be the consequence of cumulative exposures to unfavorable working conditions, poor health and low work ability.

Socioeconomic inequalities in health are well documented (4). In Germany, this applies to both physical and mental health (5). Recent studies among older workers, strengthen the assumption of causation, ie, a low SES causes poor health (6). Health in turn plays a crucial role in the retirement behavior of older workers (7–10). Poor self-perceived general health, but also poor mental and physical health have been shown to be important risk factors for premature exits from paid employment due to disability pension and unemployment (9). Consistently, the risk of a health-based selection out of employment is most pronounced for those with a low SES (7, 8).

Not only health but also certain work factors are strongly associated with SES. Socioeconomic differences are frequently reported for physical but also psychosocial demands, such as quantitative demands and job control (6, 11, 12). Several studies have examined the effect of different work factors on premature exits from employment. Most recently, a study with data from the German Study on Mental Health at Work (S-MGA) found that employees exposed to awkward body postures, heavy lifting and high work pace were at an increased risk of early exit from employment (13). An earlier study based on data from the Survey of Health, Ageing, and Retirement in Europe (SHARE) demonstrated that a lack of job control increased the risk for disability pension, unemployment, and early retirement (14).

Next to health and the working conditions, the fit of the worker’s resources and the work demands, which may be termed work ability (15), plays a crucial role in the timing of exit. Although evidence is scarce, workers with low SES seem to be at higher risk of experiencing low work ability (16, 17). Again, emerging evidence from Scandinavia, The Netherlands and most recently from Germany and the USA stress the predictive value of low work ability for early exits from work, most notably receiving disability pension (18–20), but also other exit routes, such as unemployment and inactivity prior to retirement (21).

Disability pension and unemployment are frequent early exit routes among older workers in Germany as other options, such as early old age pension, are only accessible to those aged ≥63 years with very long social security contributions (≥45 or ≥35 years with deductions) (22–25). Another possible early exit route is often termed economic inactivity and is characterized by an unspecified early labor market exit (cf, 8, 11, 25).

So far, few studies have placed focus on the contribution of work factors, health, and work ability to social inequalities in early exit from employment during the later career of older workers. Existing studies on the topic have primarily focused on a different or reduced set of mediators in the pathway of the SES and early exit from paid employment and none included work ability (8, 11, 26). Moreover, none of the studies has a specific focus on older workers in Germany. Recent advancements in causal mediation analysis (27) are well suited to analyze pathways linking the SES to early exits. The so-called inverse odds (ratio) weighting (IO[R]W) (28) is fit for non-linear regression models including survival outcomes, agnostic with regard to exposure-mediator interactions on the outcome, and can accommodate multiple mediators simultaneously (unlike other counterfactual-based approaches) (29).

Using the IOW approach, we thus aim to examine the extent to which the effect of the SES (here: education) on early exit from paid employment operates through work factors, health, and work ability.

Based on the current evidence (8, 11, 26), we assume that the relevance of these pathways varies by the type of exit route and when comparing different social strata.

## Methods

### Study design and population

We use data from the German lidA study, which is a prospective cohort study on work, age, health and labor market participation. It is representative of socially insured older employees from the German “baby boom” generation with respect to sociodemographic variables such as sex, education and occupation, covering the birth cohorts 1959 and 1965 (30, 31). A detailed description of the study design and survey methods can be found elsewhere (30, 31). Currently the study comprises three waves with a baseline measurement in 2011 (N=6585) and two follow-ups (2014, N=4244; 2018, N=3586). The survey data was linked to employment register data from the Institute for Employment Research (IAB) of the German Federal Employment Agency to enrich information on employment histories and thereby on the potential exit routes and the timing of exit. This data covers information on employment status (excluding disability) of all employees subject to social insurance, thereby excluding sworn civil servants and self-employed.

All subjects who provided written consent for the usage of their employment register data during the last available survey wave were initially included in the present study (N=2560). The follow-up period was six years (2011–2017) with annual data on exits. Subjects who entered long-term unemployment or started to receive a disability pension in or before 2011, as well as subjects who experienced an unspecified labor market exit at some point in 2011 and subjects with missing data on the main exposure variable (education; N=15) were excluded, resulting in N=2438 matching cases between the two data sources (see supplementary material, www.sjweh.fi/article/4043, figure S1 for inclusion and exclusion criteria). Thus, the analysis only included subjects who were event-free at study baseline.

### Early exit from paid employment

Three competing exit routes were defined, for which annual information was available: disability pension, long-term unemployment, and unspecified early labor market exit. Information on disability pension and unemployment events were obtained from the lidA survey data. If subjects received disability pension in 2018, they were asked to report when (year) they first entered the disability pension scheme. If subjects were unemployed in 2018, they were asked to report when (year) they left their last employment.

Information on unspecified early labor market exits was obtained from the employment register data. An unspecified early labor market exit was defined by a discontinuation of the individual’s employment history in the register data (ie, exit from social insurance/gap spell). Only if subjects spent most of at least one year in this status (modal state), they were assigned this exit.

The time to the first event was recorded. Register data was available until 2017. Subjects with no event after exposure measurement in 2011 until the end of the follow-up period (2012–2017) were censored.

### Socioeconomic status

SES was operationalized by the level of education, using a combined score of education and vocational training (32). The score was subsequently categorized into three classes of low (primary, lower secondary and upper secondary general education, cf. ISCED-97 1–3A), moderate (upper secondary vocational education and post-secondary non tertiary education, cf. ISCED-97 3B–4A), and high education (tertiary education, cf. ISCED-97 5–6).

### Work factors

In total, we assessed three work factors which, based on current evidence (6, 11–14), seemed to be plausible mediators between the SES and early exit from employment: physical demands, quantitative demands, and influence at work (as a proxy of job control). Physical demands were assessed with three items measuring the time exposed to awkward body postures, heavy lifting, and repetitive movements. Subjects exposed to any of the three dimensions for >25% of the working time were regarded as having high physical demands. Quantitative demands (low/high) and influence at work (low/high) were measured with items from the German version of the Copenhagen Psychosocial Questionnaire (COPSOQ) (33). The scales have been validated and show good psychometric properties (33). The resulting sum scores for each domain were subsequently dichotomized at the median.

### Health

Physical and mental health were assessed using the Short Form Health Survey (SF-12) (34). Two separate sum scores were calculated for both health scales following Nübling, Andersen and Mühlbacher (35). Subsequently, they were dichotomized at the median, dividing the sample into groups of less than good versus good (mental or physical, respectively) health.

### Work ability

The second dimension of the Work Ability Index (WAI2) (15) was used to parameterize work ability. WAI2 assesses the work ability in relation to mental and physical work demands with two items. A third item measuring whether subjects are mainly mentally or physically active in their jobs was used to weigh the responses to the first two items. The resulting sum score (2 [no work ability] to 10 [high work ability]) was dichotomized. The cut-off point was set at 8, defining subjects with low (<8) and high (≥8) work ability. Ebener & Hasselhorn (36) validated the short measure and illustrated the advantages of its application in occupational health research.

### Confounding variables

Age was assessed by the participants’ birth year, resulting in two groups aged 46 (born 1965) or 52 (born 1959) at baseline (2011). Sex (male/female) and partner status (partnership/single) were dichotomous.

All covariates including the main exposure education were assessed at baseline in 2011. Since all included subjects were aged 46 or 52 at entry, it can be assumed that education preceded their work factors, health, and work ability at the time of the study.

### Statistical analysis

Firstly, descriptive statistics were used to present the baseline characteristics of the study population and the number of early exits during the 6-year follow-up by educational level (table 1). Secondly, we estimated the main effects of the SES and the potential mediators on early exit from employment using Cox proportional hazard analyses (table 2). The proportional hazard assumption was checked based on Schoenfeld residuals (37). A respective P>0.05 indicates that the assumption of proportional hazards holds.

**Table 1:** Baseline characteristics of the study sample and labor force exit during the 6-year follow-up (N=2438 ^a^).

Characteristics	Educational level			

	Low (N=509)	Moderate (N=1 394)	High (N=535)	Data missing
			
	N (%)	N (%)	N (%)	N (%)
Sex				0
Female	229 (44.9)	852 (61.1)	247 (46.2)	
Male	280 (55.1)	542 (38.9)	288 (53.8)	
Age at entry (2011)				0
46 (born 1965)	271 (53.2)	816 (58.5)	304 (56.8)	
52 (born 1959)	238 (46.8)	578 (41.5)	231 (43.2)	
Partner status				7 (0.3)
Single	71 (13.9)	168 (12.1)	50 (9.4)	
Not single	438 (86.1)	1 221 (87.9)	483 (90.6)	
Work factors				
High physical demands	292 (58.5)	692 (50.6)	204 (38.7)	43 (1.8)
High quantitative demands	181 (36.2)	678 (49.5)	302 (57.1)	40 (1.6)
Low influence at work	240 (48.1)	593 (43.3)	129 (24.4)	41 (1.7)
Health				
Less-than-good (physical)	308 (61.7)	686 (50.3)	175 (33.2)	49 (2.0)
Less-than-good (mental)	248 (49.7)	652 (47.8)	249 (47.3)	49 (2.0)
Work ability				49 (2.0)
Low	193 (38.8)	429 (31.5)	117 (22.1)	
Labor force exit (2011–2017)				0
Disability pension	23 (4.3)	36 (2.6)	5 (0.9)	
Unemployment	19 (3.7)	34 (2.4)	7 (1.3)	
Unspecified	19 (3.7)	46 (3.3)	21 (3.9)	
Censored	448 (88.3)	1 275 (91.7)	502 (93.8)	

a Valid column percentages displayed.

**Table 2:** Main effects of socioeconomic status (SES), work factors, health, and work ability on the likelihood of early exit during a 6-year follow-up. Cox Proportional Hazard Regression: for each exit route, the independent variables were entered separately into the regression model; all models were adjusted for age, sex, partner status; statistically significant hazard ratios (HR) (P<0.05) with 95% confidence interval (CI) marked in bold; the proportional hazard assumption was fulfilled for all models.

	Disability pension	Unemployment	Labor market exit
		
	HR (95% CI)	HR (95% CI)	HR (95% CI)
SES (education) (N=2431)			
Low vs moderate ^a^	**1.83 (1.08–3.13**)	1.69 (0.96–2.98)	1.15 (0.67–1.98)
Low vs high ^b^	**4.62 (1.75–12.22)**	**2.85 (1.20–6.79)**	0.98 (0.52–1.80)
Work factors ^c^ (N=2387)			
Physical demands			
High	1.48 (0.87–2.53)	1.61 (0.92–2.84)	0.94 (0.61–1.46)
Quantitative demands			
High	0.97 (0.58–1.63)	1.57 (0.90–2.74)	1.06 (0.69–1.63)
Influence at work			
Low	1.03 (0.61–1.76)	0.70 (0.39–1.25)	0.86 (0.54–1.36)
Health (N=2383)			
Physical health			
Less than good	**1.90 (1.10–3.27)**	**2.79 (1.51–5.14)**	1.02 (0.66–1.57)
Mental health			
Less than good	**2.23 (1.29–3.87**)	1.51 (0.87–2.61)	1.09 (0.70–1.68)
Work & Worker (N=2383)			
Work ability			
Low	**4.44 (2.55–7.71)**	1.70 (0.99–2.94)	1.33 (0.85–2.08)

a Moderate education as reference, effect estimates for high vs moderate not displayed.

b High education as reference, effect estimates for moderate vs high not displayed.

c Variables from the work domain were mutually adjusted.

**Figure 1:**
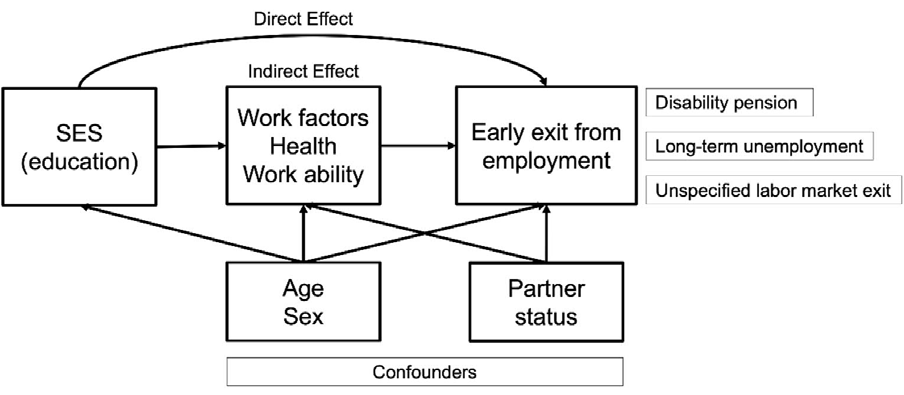
Hypothesized relationship between education, work factors, health, work ability and early exit from employment.

To examine the mediating effects of work, health, and work ability we applied an IOW approach to decompose the total effect (TE) of education on the outcome (ie, the survival time to the first exit event) into a natural direct effect (NDE) and natural indirect effect (NIE) (28, 29) (figure 1, tables 3–4). The NDE captures the effect of education on the outcome if the pathway through the mediator of interest was disabled, NIE the effect of education through the intermediate and TE the sum of NDE and NIE (38). The definitions of these effects estimates are based on the counterfactual framework (38, 39). We compared groups with low versus high and low versus moderate educational level.

**Table 3:** Decomposition of the effect of socioeconomic status (SES) on disability pension into total effect (TE), natural direct effect (NDE) and natural indirect effect (NIE) using work factors, health, and work ability as mediators (N=2438). TE and significant NIE and respective proportion mediated (PM) marked in bold. Adjusted for age, sex, partner status. [BC=bias corrected; CI=confidence interval; HR=cause-specific hazard ratio.]

	Low versus high SES ^a^			Low versus moderate SES ^a^		
	
	HR	BC CI ^b^	PM ^c^%	HR	BC CI ^b^	PM ^c^%
Analysis 1: Physical demands						
NIE	1.01	0.91–3.62	1	1.03	0.93–1.22	7
NDE	4.57	1.81–16.79		1.77	1.02–3.21	
Analysis 2: Quantitative demands						
NIE	0.88	0.73–1.06	–17	0.88	0.70–1.05	–30
NDE	5.25	2.18–23.36		2.08	0.99–3.59	
Analysis 3: Influence at work						
NIE	1.03	0.87–1.15	4	1.04	0.88–1.17	8
NDE	4.49	1.66–16.91		1.76	0.94–3.02	
Analysis 4: Physical health						
NIE	1.04	0.83–1.24	5	1.06	0.84–1.27	12
NDE	4.45	1.82–22.79		1.73	0.95–3.00	
Analysis 5: Mental health						
NIE	1.01	0.89–1.09	1	1.02	0.89–1.12	4
NDE	4.59	1.92–23.54		1.80	1.04–3.24	
Analysis 6: Work ability						
NIE	**1.22**	**1.07–3.99**	**23**	**1.21**	**1.06–1.50**	**38**
NDE	3.80	1.50–15.41		1.51	0.87–2.54	
Analysis 7: Health & work ability						
NIE	**1.27**	**1.06–1.76**	**27**	**1.27**	**1.03–1.64**	**47**
NDE	3.65	1.49–16.49		1.44	0.78–2.51	
Analysis 8: Health & work ability & work factors ^d^						
NIE	1.12	0.87–4.18	14	1.11	0.86–1.76	22
NDE	4.13	1.64–24.01		1.65	0.79–3.01	
TE of SES ^a^	**4.62**	**1.88–21.07**		**1.83**	**1.06–3.19**	

a Educational level.

b Obtained from bootstrapping (1000 reps)

c Proportion mediated = HR_NDE_*(HR_NIE_-1)/(HR_NDE_*HR_NIE_-1)

d 2.4% smaller sample size in fully adjusted multi-mediator model due to listwise exclusion of missing values (N=2 380)

**Table 4:** Decomposition of the effect of the socioeconomic status (SES) on unemployment into total effect (TE), natural direct effect (NDE) and natural indirect effect (NIE) using work factors, health, and work ability as mediators (N=2438). TE and significant NIE and respective proportion mediated (PM) marked in bold. Adjusted for age, sex, partner status. [BC=bias corrected; CI=confidence interval; HR=cause-specific hazard ratio.]

	Low versus high SES ^a^			Low versus moderate SES ^a^		
	
	HR	BC CI ^b^	PM ^c^%	HR	BC CI ^b^	PM ^c^%
Analysis 1: Physical demands						
NIE	1.29	0.99–1.59	35	**1.30**	**1.07–1.52**	**57**
NDE	2.20	0.79–6.92		1.30	0.64–2.55	
Analysis 2: Quantitative demands						
NIE	1.03	0.87–1.20	5	1.04	0.86–1.22	9
NDE	2.75	1.06–8.40		1.63	0.75–2.87	
Analysis 3: Influence at work						
NIE	**1.22**	**1.05–1.38**	**28**	**1.23**	**1.04–1.42**	**46**
NDE	2.34	0.89–6.90		1.37	0.64–2.65	
Analysis 4: Physical health						
NIE	**1.33**	**1.25–2.23**	**38**	**1.32**	**1.23–1.76**	**59**
NDE	2.15	0.80–5.95		1.28	0.58–2.47	
Analysis 5: Mental health						
NIE	**1.22**	**1.07–1.37**	**28**	**1.22**	**1.06–1.37**	**44**
NDE	2.34	0.96–6.59		1.39	0.69–2.56	
Analysis 6: Work ability						
NIE	**1.29**	**1.10–1.48**	**35**	**1.28**	**1.08–1.48**	**54**
NDE	2.21	0.92–7.13		1.32	0.63–2.56	
Analysis 7: Health & work ability						
NIE	**1.39**	**1.26–3.76**	**43**	**1.36**	**1.25–1.95**	**65**
NDE	2.05	0.85–7.75		1.24	0.58–2.47	
Analysis 8: Health & work ability & work factors ^d^						
NIE	**1.28**	**1.08–2.12**	**34**	**1.28**	**1.09–2.13**	**54**
NDE	2.23	0.79–7.83		1.32	0.55–2.52	
TE of SES ^a^	**2.85**	**1.38–12.99**		**1.69**	**1.14–3.49**	

a Educational level.

b Obtained from bootstrapping (1000 reps)

c Proportion mediated = HR_NDE_*(HR_NIE_-1)/(HR_NDE_*HR_NIE_-1)

d 2.4% smaller sample size in fully adjusted multi-mediator model due to listwise exclusion of missing values (N=2380)

In line with previous studies applying the IOW approach (12, 29, 40), the IOW estimation of NDE and NIE consisted of the following steps. First, a multinomial regression model was fitted for education conditional on the mediator(s) and the confounding variables. Second, the individual’s IOW was calculated by taking the inverse of the predicted odds from the first step. The reference group (first the high educated, then the moderate educated) was assigned with a weight equal to 1. Third, the TE was estimated by using a cause-specific Cox regression model, regressing each exit route separately on education and the confounding variables. For this step, we declared the data to be survival-time data with Stata’s “stset” command (37) without specifying a weight. Fourth, the NDE was estimated using the same model, but specifying the weights that were assigned to each subject in step 2. Lastly, the NIE was obtained by subtracting the NDE from the TE. We used bootstrapping with 1000 replications to derive the effect estimates and bias-corrected confidence intervals (CI) (41). Bias-corrected CI not including 1 indicate statistically significant effects and yield better coverage probabilities than normal approximation CI if the bootstrap distribution deviates from normal (41). A P<0.05 was considered statistically significant. Missing values of single variables did not exceed 5%. Hence, missing data were handled by listwise exclusion. The percentage of cases excluded did not exceed 2.4% in fully adjusted multi-mediator models. We calculated the proportion mediated (PM) using VanderWeele’s (38) equation for ratio measures: HR_NDE_*(HR_NIE_-1)/(HR_NDE_*HR_NIE_-1). In order to compute the NDE and NIE we assumed the absence of unobserved confounding for (i) the exposure-outcome relationship, (ii) for the mediator-outcome relationship and (iii) for the exposure-mediator relationship. Additionally, the absence of (iv) a mediator-outcome confounder that is an effect of the exposure was assumed (29). All analyses were conducted using Stata V15.1 (StataCorp LLC, College Station, TX, USA).

### Sensitivity analysis

We conducted a competing risk regression using the Fine & Gray (42) model, estimating the main effects of education and the covariates on the competing exit routes to assess whether using this model would have changed the estimates compared to the Cox model.

### Ethical approval

The Ethics Committee of the University of Wuppertal approved the protocol for the lidA Cohort study [5 December 2008 (Sch/Ei Hasselhorn) and 20 November 2017 (MS/BB 171025 Hasselhorn)]. All participants were informed about the aims and procedures of the study. In accordance with data protection requirements, verbal consent was required for participation at baseline and for each follow-up wave and written consent was required for data linkage.

## Results

### Main findings

We found a higher prevalence of high physical demands, low influence at work, less-than-good physical health and low work ability among subjects with low SES (table 1). High quantitative demands were more prevalent in groups with higher SES (table 1, see supplementary table S2 for the strength of these associations). Furthermore, a larger proportion of subjects with low SES (4.3%) started to receive disability pension when compared to moderate (2.6%) or high (0.9%) SES groups. This similarly applied to becoming long-term unemployed during follow-up time, while no such social gradient was apparent with respect to unspecified premature labor market exits (table 1). In total, 11.7% exited employment early in the low SES group, 7.3% in the moderate SES group and 6.2% in the high SES group.

When compared to subjects with a high SES, employees with a low SES had a more than four-fold instantaneous rate of exiting into disability pension (HR 4.62, 95% CI 1.75–12.22) and an almost three-times higher rate of becoming long-term unemployed (HR 2.85, 95% CI 1.20–6.79) during the 6-year follow-up (table 2). Also, when compared to subjects with a moderate SES, those with a low SES had a significantly higher instantaneous rate of exiting into disability pension (HR 1.83, 95% CI 1.08–3.13) and a borderline significantly higher instantaneous rate of exiting into unemployment (HR 1.69, 95% 0.96–2.98) (table 2). Neither the SES nor any potential mediator exerted a statistically significant effect on unspecified labor market exit and hence mediation analysis was not conducted for this outcome. For all analysis models, the proportional hazard assumption was fulfilled (ph-test P-value >0.05).

Table 3 shows the effect decomposition of the TE of the SES on disability pension into a NIE and NDE using work factors, health, and work ability as mediators. When comparing low versus high SES groups, 23% of the effect of the SES on disability pension was mediated by low work ability. Taking health and low work ability together, these factors explained 27% of the social inequalities in early exits into disability pension comparing low to high SES groups. Social inequalities between groups of low and moderate SES could be explained to an even higher degree by work ability (38%) and by health and work ability combined (47%).

When investigating unemployment as the outcome (table 4), (borderline) significant NIE were observed for all mediators except quantitative demands. We found that physical health mediated the largest proportion (38%) of the effect of the SES on this exit route when comparing low versus high SES groups, followed by work ability (35%), mental health and influence at work (each 28%). Estimating the mediation effects of health and work ability combined, the PM was 43%. The PM fell to 34% when work factors were added (table 4, analysis 8). A similar pattern was observed comparing low versus moderate SES groups: 59% of the effect of the SES on long-term unemployment was explained by physical health, followed by physical demands (57%), work ability (54%), influence at work (46%) and mental health (44%). The combined mediation effect of health and work ability lead to a PM of 65%.

### Sensitivity analysis

Using the Fine & Gray (F&G) competing risk regression to determine the main effects of education and the covariates on the probability of leaving employment through one of the competing exit routes did not reveal considerable differences compared to the Cox model (supplementary table S3). We note that the sub-distribution HR from the F&G model are not directly comparable to the HR as they are on a different scale.

## Discussion

### Main findings

Among older workers, those with a low SES (operationalized by education) had an increased risk of exiting employment early through disability pension and by entering long-term unemployment but not through an unspecified premature labor market exit. The results suggest that for older workers in Germany, low work ability may be the most important pathway through which the SES exerts its effect on employment exits through disability pension, accounting for 23% of the social inequalities between low and high SES groups, and 38% between low and moderate SES groups.

With respect to becoming long-term unemployed, the effect of the SES on this exit route operates to a large extent through poor physical health, which accounts for 38% of social inequalities comparing low to high SES groups and 59% comparing low to moderate SES groups. The combined analyses of health and work ability consistently lead to the highest PM values. As much as 65% of the effect of the low versus moderate SES on long-term unemployment was mediated by the combination of health and work ability.

The study indicates that work factors, health, and work ability explain social inequalities in early exit from employment between low and moderate SES groups to a larger extent than the social inequalities between low and high SES groups.

### Comparability with existing evidence

Our findings coincide with previous analyses by Robroek et al (11) and Schuring et al (8), who demonstrated that lower educated workers had an increased risk of leaving paid employment due to disability benefits and unemployment.

In our study, work ability turned out to be the main contributor to social inequalities in early exit through disability pension, while work factors and health played a minor role. Our findings thereby deviate from a similar study by Robroek et al (11), who found that self-perceived health mediated large parts of the effect of the SES on disability pension. Work ability was not investigated in that study. We assume two main causes of the predominant role of work ability and the minor role of health in our study. Firstly, continuing to work may be possible despite poor health, but difficult in the presence of low work ability. In a recent discussion paper (43), the authors stress that older workers with poor health may continue working if they have to for financial reasons, an argument applying specifically to those with low SES. Conversely, those experiencing low work ability may have no other option than to exit employment. This assumption is supported by existing evidence indicating a strong predictive value of low work ability for subsequent disability pension (18–20).

Secondly, in Germany, eligibility criteria for access to disability pension are formally based on the work ability of employees. Disability pension is not granted unless workers are incapable of working in any kind of job for more than three (full disability pension) or six hours per day (partial disability pension) (25).

When it comes to long-term unemployment, in our analysis health, work ability, and work factors significantly contributed to social inequalities in early exits. Physical health was the dominating mediator, with a proportion mediated of 38% between low and high SES groups. In a similar study (11), the mediating effect of self-perceived health on unemployment was smaller (9%). The differences between the studies may not only be explained by the different operationalization of health, but also by the age of the analysis samples. While Robroek et al (11) investigated workers aged 18–64 years, our sample consists of workers aged 46 and 52 years at baseline. The effect of poor health on exits into unemployment may be more pronounced for older workers, especially in the presence of unfavorable working conditions (44, 45).

Lastly, work factors, health, and work ability consistently explained social inequalities in early exits between low and moderate SES groups to a larger degree than inequalities between low and high SES groups. Comparable observations were made in a methodologically similar study investigating the outcome health among older workers (12). In our study, the set of investigated mediators mainly reflects aspects of the work and health domains. However, those with a high SES might differ from those with lower SES with respect to many further life aspects with potential influence on labor market participation. Eg, existing evidence suggests that those with higher SES also have a healthier lifestyle (11) and more stable employment relationships (46), both protective of early exit from employment.

### Strengths and limitations

To our knowledge, this is the first study examining social inequalities in early exit from paid employment applying an IOW approach with discrete survival time data. An important strength of the IOW approach is that a causal interpretation of the effect estimates is possible irrespective of a potential exposure-mediator interaction (29).

A further strength of the study is the linkage between survey data and employment register data, whereby annual information on three early exit routes could be obtained. However, we would like to stress some limitations inherent with the longitudinal study design.

Compared to the initial lidA study sample, lower educated subjects (26% at wave 1) are slightly underrepresented in the current analysis sample (21%). The total effects of the SES on the three exit routes may therefore be underestimated, if lower educated are more likely to exit early, as similar studies indicate (11, 46). Additionally, a healthy worker effect may contribute to an underestimation of the mediation effects, especially of health variables.

We also note that for some analyses relatively few events per independent variable were observed. This mainly applies to the fully adjusted multi-mediator models, where less than the recommended ten events per independent variable for Cox regression models (47) were reached. Thus, the respective effect estimates need to be interpreted with caution.

A strength of this study is the focus on few age groups as the mechanisms leading to early exit and potentially to social differences in early exit may be assumed to be age related (3). This, however, limits the external validity of the results for other age groups (see above). Furthermore, the country-specific context regarding early exits may hamper the generalizability of our findings to non-German contexts (48).

Lastly, the causal interpretation of the NDE and NIE is based on the no-unmeasured confounding assumptions, which were formulated above. Leaving employment is a complex process (2). Although we adjusted for common outcome-mediator and mediator-outcome confounders, residual confounding cannot be ruled out. Concerning assumption 4, only the partner status is post-exposure and may violate the assumption. However, our analysis indicated that this mediator-outcome confounder is not statistically significantly associated with the exposure.

### Implications

In times of extended working life policies, our findings highlight the necessity of investments into selective prevention of low work ability and poor physical health among (older) workers to forestall a widening of social inequalities in early exits through disability pension and long-term unemployment. So far, in many countries, extended working life policies do not sufficiently consider groups of workers with different needs and resources as well as cumulative (dis-)advantages over the life course (2).

The findings further implicate that preventive measures targeting these intermediates may be more effective leveling inequalities between the low and moderate SES groups and – to a much lesser extent – those between low and high SES groups. Differences in employment participation between older workers with low and high SES may thus be explained to a larger extent by a conglomeration of further unobserved factors. Future studies should make use of the advantages of the IOW approach to examine an even broader selection of intermediates collectively, including health behavior and employment arrangements.

### Concluding remarks

Our findings indicate low work ability and less-than-good physical health may propel social inequalities in early exit from paid employment through disability pension and long-term unemployment among older workers. Work factors contributed considerably to social inequalities in exits through unemployment but not through disability pension. Current extended working life policies should be accompanied by preventive measures addressing these factors to counteract the increase of social inequalities during the later career of older employees.

## Supplementary material

Supplementary material
