# Supplementary material for: Social inequalities in early exit from employment in Germany: a causal mediation analysis on the role of work, health, and work ability
Source: Scand J Work Environ Health. 2022 Oct 1;48(7):569–78. doi: 10.5271/sjweh.4043 (PMC10539108; doi:10.5271/sjweh.4043)
Supplement: Supplementary material [file SJWEH-48-569-S001.pdf]

Social inequalities in early exit from employment in Germany: a causal mediation analysis on the role of work, health, and work ability[1]

by Max Rohrbacher, MSc,[2] Hans Martin Hasselhorn, MD

1. Supplementary Tables/Figures
2. Correspondence to: Max Rohrbacher, Department of Occupational Health Science, School of Mechanical Engineering and Safety Engineering, University of Wuppertal, Gaussstrasse 20, 42119 Wuppertal, Germany. [E-mail: rohrbacher@uni-wuppertal.de]

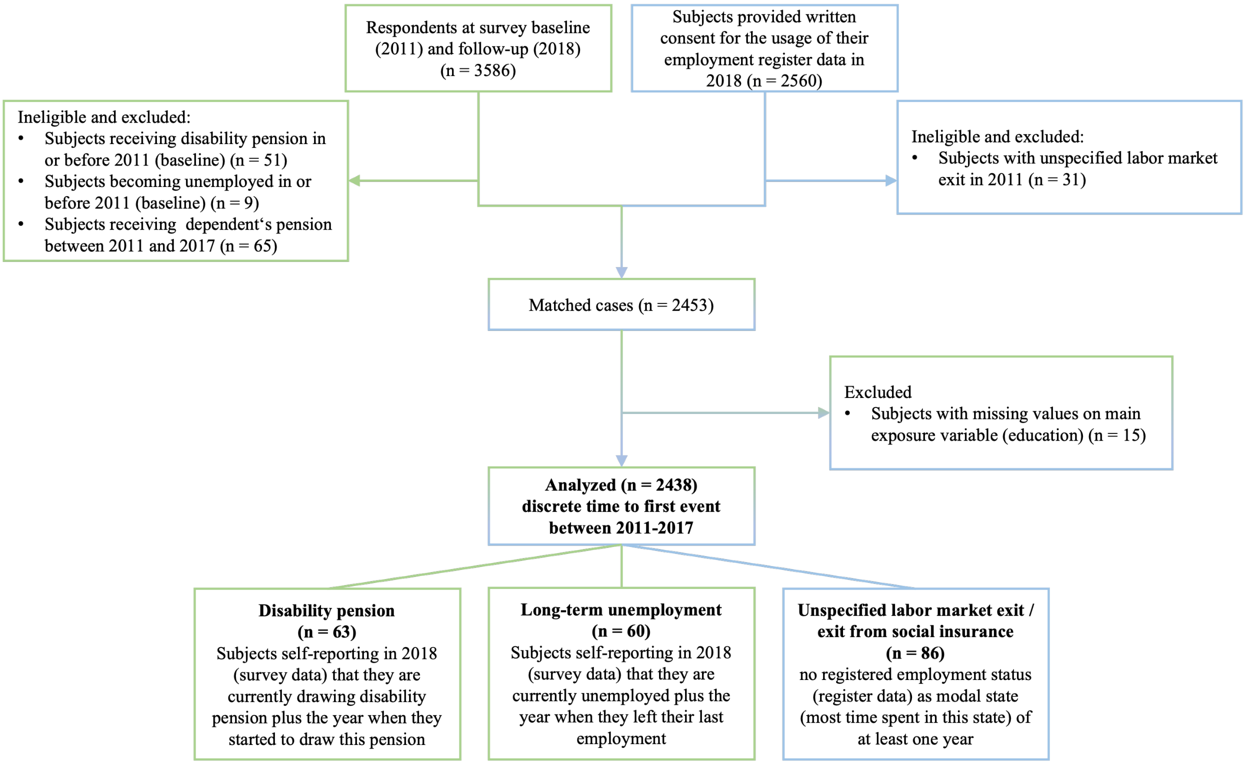

Supplementary figure S1. Flow diagram showing inclusion and exclusion criteria

Supplementary table S2. Bivariate association between education and work factors, health, and work ability at study baseline (2011) (N=2 438). Logistic Regression

		High education		Moderate education		Low education	
		%	OR (95%CI)	%	OR (95%CI)	%	OR (95%CI)
**Work factors**							
Physical demands	high	39	**0.45 (0.35-0.58)**	51	**0.71 (0.58-0.88)**	59	1.00
Quantitative demands	high	57	**2.37 (1.84-3.04)**	50	**1.77 (1.43-2.19)**	36	1.00
Influence at work	low	24	**0.34 (0.26-0.45)**	43	**0.76 (0.62-0.94)**	48	1.00
**Health**							
Physical Health	Less than good	33	**0.31 (0.24-0.40)**	50	**0.63 (0.51-0.77)**	62	1.00
Mental Health	Less than good	47	0.91 (0.71-1.17)	48	0.90 (0.73-1.11)	50	1.00
**Work & Worker**							
Work ability	low	22	**0.45 (0.34-0.59)**	32	**0.71 (0.57-0.88)**	39	1.00

Adjusted for age, sex, and partner status
Logistic Regression conducted separately for each covariate
Statistically significant ORs and 95% CIs in bold

Supplementary table S3. Influence of the SES, work factors, health, and work ability on the likelihood of early exit during a 6-year follow-up among older employees in Germany. Competing Risk Regression [SHR = subdistribution hazard ratio]

		Disability pension	Unemployment	Labor market exit
		SHR (95%CI)	SHR (95%CI)	SHR (95%CI)
**SES (education)** (n=2431)	Low vs moderate[b]	**1.81 (1.07-3.08)**	1.67 (0.93-2.95)	1.14 (0.66-1.98)
	Low vs high[c]	**4.58 (1.72-12.16)**	**2.78 (1.14-6.77)**	0.95 (0.51-1.76)
**Work factors**[a] (n=2387)				
Physically demands	high	**1.47 (0.87-2.49)**	1.60 (0.92-2.79)	0.93 (0.61-1.44)
Quantitative demands	high	0.96 (0.57-1.62)	1.57 (0.90-2.74)	1.06 (0.69-1.62)
Influence at work	low	1.03 (0.61-1.76)	0.70 (0.40-1.24)	0.86 (0.56-1.33)
**Health** (n=2383)				
Physical health	less than good	**1.88 (1.10-3.20)**	**2.74 (1.50-5.02)**	1.01 (0.66-1.56)
Mental health	less than good	**2.23 (1.30-3.84)**	1.47 (0.85-2.55)	1.07 (0.70-1.65)
**Work & Worker** (n=2383)				
Work ability	low	**4.37 (2.52-7.59)**	1.63 (0.95-2.81)	1.30 (0.83-2.04)

For each exit route the independent variables were entered separately into the regression model
All models were adjusted for age, sex, partner status; statistically significant SHRs ($p < 0.05$) with 95% CIs marked in bold.
[a]Variables from the work domain were mutually adjusted
[b]high education as reference, effect estimates for moderate vs high not displayed
[c]moderate education as reference, effect estimates for high vs moderate not displayed
